# The Integration of Biology Into the Treatment of Diffuse Intrinsic Pontine Glioma: A Review of the North American Clinical Trial Perspective

**DOI:** 10.3389/fonc.2018.00169

**Published:** 2018-05-18

**Authors:** Jessica Clymer, Mark W. Kieran

**Affiliations:** ^1^Department of Pediatric Oncology, Dana-Farber Cancer Institute, Boston, MA, United States; ^2^Division of Pediatric Hematology/Oncology, Boston Children’s Hospital, Boston, MA, United States; ^3^Harvard Medical School, Boston, MA, United States

**Keywords:** diffuse intrinsic pontine gliomas, brainstem glioma, targeted therapy, convection-enhanced delivery, immunotherapy, clinical trials

## Abstract

Dramatic advances in the molecular analysis of diffuse intrinsic pontine glioma have occurred over the last decade and resulted in the identification of potential therapeutic targets. In spite of these advances, no significant improvement in the outcome has been achieved and median survival remains approximately 10 months. An understanding of the approaches that have been taken to date, why they failed, and how that information can lead the field forward is critical if we are to change the *status quo*. In this review, we will discuss the clinical trial landscape in North America with an overview of historical approaches that failed and what might account for this failure. We will then provide a discussion of how our understanding of the genotype of this disease has led to the development of a number of trials targeting the mutations and epigenome of diffuse intrinsic pontine gliomas and the issues related to these trials. Similarly, the introduction of methodologies to address penetration across the blood–brain barrier will be considered in the context of both targeted approaches, epigenetic modification, and immune surveillance of these tumors. The comprehensive analysis of these data, generated through cooperative groups, collaborative clinical trials, and pilot studies in North America will be the focus of the IVth Memorial Alicia Pueyo international symposium in Barcelona on March 12th, 2018 and will be compared and contrasted with a similar comprehensive analysis of the European data with the goal of bringing all of these data together to develop a uniform platform on which new rational trials can be based.

## Introduction

Diffuse-intrinsic pontine gliomas (DIPG) are the most common brainstem tumors in children and remain the deadliest cancer diagnosis in this population. It occurs in all age groups but is most commonly seen in children between the ages of 5–10 years with an equal distribution between the sexes (M:F = 1:1). DIPG remains one of the most challenging of all pediatric cancers and its outcomes remain abysmal. Median progression-free survival continues to range from 5 to 9 months with overall survival at 6–16 months despite hundreds of clinical trials.

## Clinical Presentation and Diagnosis

Historically, DIPG has been a clinical-radiographic diagnosis as biopsy and thus histopathologic confirmation was deemed unsafe and did not influence either treatment or outcome. Patients with DIPG often have a short latency (<3 months) between symptom onset and diagnosis. At time of diagnosis, acute symptoms include cranial neuropathies, long tract signs, and ataxia with a minority (<10%) of patients presenting with symptoms of raised intracranial pressure. Classic radiographic features on MRI include a T1 hypointense T2 hyperintense mass occupying more than 50% of the pons, causing expansion of the pons, and often encircling the basilar artery. It appears to obey the pontomedullary boundary. Post gadolidium enhancement can be variable from rim enhancement to patchy enhancement to complete absence of enhancement. When present, contrast enhancement is a poor prognostic indicator (1). The presence of these imaging characteristics coupled with acute symptoms has long been sufficient for the diagnosis and treatment of children with DIPG. However, with a growing utilization of biopsies at diagnosis, a new WHO classification applicable to some DIPG tumors has emerged—diffuse midline glioma, H3K27M mutant (discussion to follow).

## Standard Treatment of DIPG

The backbone of treatment for children with newly diagnosed DIPG continues to be focal, wide field radiation therapy to the pons. Most centers utilize 3D conformational photon-based radiotherapy to 54–59.4 Gy given in 30–33 fractions of 1.8 Gy daily. Radiation therapy may allow for relief of neurologic symptoms in most patients and a reduction or cessation of systemic steroids for many. However, radiation therapy overall is seen as a means of palliation, taking the overall survival from weeks to months. Increasing the dose of RT using hyper-fractionated protocols (66–78 Gy) does not appear to provide a survival benefit when compared to standard dose and fractionation protocols (2–7). Some international groups have sought to demonstrate the feasibility of hypo-fractionated radiation therapy in an effort to improve palliation (8–10), and thus the ideal fractionation strategy continues to cause some debate.

## Radiation Sensitizer Clinical Trails

The only improvement to date in the outcome of DIPG has been the addition of radiation therapy. A rational approach meant to build on this impact has been to add agents that can sensitize or synergize the effects of radiation therapy. The majority of these have focused on improving areas of hypoxia so that the concentration of oxygen radicals needed for the radiation effect can be achieved. While multiple approaches have been tried and some drugs entered pediatric clinical trials (11), none have improved the median or overall survival and most have been associated with a significant increase in toxicity (12).

## Chemotherapy Clinical Trials

Various chemotherapeutic strategies have been used to treat patients with DIPG including neo-adjuvant chemotherapy, concurrent chemotherapy with RT, adjuvant chemotherapy, and high dose myeloablative chemotherapy with stem cell rescue (13–16). Unfortunately, none have demonstrated improved survival when compared to radiation therapy alone. In particular, the standard of care for adult glioblastoma, which includes radiation therapy with concurrent and adjuvant temozolomide has not been shown to benefit newly diagnosed DIPG patients (17–19). While small series have indicated some effect of antiangiogenic therapy with bevacizumab (20), by and large this therapy has failed to markedly improve survival in both the newly diagnosed and relapse setting (21, 22). While a multitude of experimental agents and chemotherapy regimens have been utilized in DIPG, many of these trials specifically accrued patients with recurrent disease. Given the often short latency from disease recurrence to death, testing agents in this setting may have decreased the likelihood of finding active agents.

## Biologic Advances

The lack of treatment progress despite decades of attempts highlighted a lack of understanding of the biologic underpinnings of this devastating disease. In years past, those pretreatment specimens that were available were often atypical cases that had required biopsy and, therefore, may not appropriately reflect typical DIPG biology. Across many centers, a concerted effort was made to obtain postmortem specimens from DIPG patients, which yielded many new insights. However, postmortem specimens are inherently limited as there may be significantly different molecular characteristics between the primary untreated tumor and the posttreatment postmortem tumor due to the selective pressure of radiation and chemotherapy. Given the limitations of postmortem samples, renewed interest in obtaining pretreatment specimens of DIPG bloomed. Large centers were able to demonstrate that surgical biopsy of DIPG at the time of diagnosis was safe and feasible and yielded sufficient sample for meaningful analysis with low morbidity (23–26).

The advent of diagnostic biopsies in DIPG has provided adequate tumor tissue for genome and epigenome sequencing. This revealed recurrent somatic mutations of *H3F3A* and *HIST1H3B* resulting in lysine 27 to methionine (K27M) substitution in the encoded histone H3.3 or H3.1. These mutations have been shown to be gain-of-function mutations that alter the polycomb repressive complex 2 leading to aberrant gene expression and thus drives cell transformation (27). Mutations in these histones, the proteins which package DNA into chromatin, have been found in approximately 80% of DIPG (28, 29). This consistent finding led to a revision in the WHO classification of CNS tumors—diffuse midline glioma, H3K27M-mutant (30)—for tumors once referred to as DIPG. This change has important implications as new clinical trials increasingly focus on the 80% of DIPG with histone mutations (and often include thalamic H3K27M mutated tumors, the biology of which may not be identical to classical DIPG). Similarly, the 20% of classic DIPG that lack histone mutations are still DIPG. When reviewing clinical trial data between studies, it will be important to recognize the different population in molecularly classified from radiographically classified DIPG. Equally important is the growing recognition of the heterogeneity in median survival between the different genomic variants of DIPG (31, 32) something that will have to be taken into account as new treatments are compared to historical controls.

## Surgical Therapeutic Approaches

An important component of DIPG resistance to traditional chemotherapy is thought related to the blood–brain barrier, which is more impermeable at the pons. Thus, strategies to bypass the blood–brain barrier have been developed (33). One such method is convection-enhanced delivery (CED) whereby catheters are placed stereotactically into the tumor and drugs administered through these small catheters directly into the tumor. The feasibility of this technique was first demonstrated in the 1990s on small animals (34), followed by animal studies to demonstrate the feasibility of this delivery method into the brainstem (35). More recently, brainstem CED has been used safely on a limited clinical basis both outside and within the context of clinical trials (36–39). CED approaches now include both multi-catheter devices, which allow for the coverage of different areas within the tumor, as well as single catheter approaches that can be re-implanted for repeated infusion.

In addition to CED, others have proposed that intra-arterial chemotherapy administration may be advantageous when compared to systemic chemotherapy for intracranial neoplasms (40). There has been significant effort to disrupt the blood–brain barrier in conjunction with intra-arterial chemotherapy administration (41, 42). These techniques have been used in combination in two cases of brainstem lesions *via* basilar artery administration (43, 44). While there have been multiple attempts to use blood–brain barrier agents in combination with traditional chemotherapy, these have not generated improved outcomes although significant worsening of toxicity was often observed (45).

## Current Strategies

Building upon previous decades’ advances in understanding of DIPG as an entity distinct from adult GBM, new clinical trials seek to further understand and exploit recently discovered molecular underpinnings of this challenging diagnosis. Table 1 provides a current list of North American trials for newly diagnosed DIPG (upper part of table), newly diagnosed DIPG after radiation therapy (middle part of table), and recurrent/progressive DIPG (bottom part of table). Clinical trials from Europe will be detailed in a different manuscript in this edition.

**Table 1:** Active North American clinical trials for children with diffuse intrinsic pontine gliomas (DIPG) as found on www.Clinicaltrials.gov as of March 1, 2018.

Current North American Trials in DIPG			
NCT Number	Title	Intervention	Site
**Newly diagnosed DIPG trials**			

NCT00879437	Valproic Acid and Radiation followed by maintenance valproic acid and bevacizumab in children with high grade gliomas or diffuse intrinsic pontine glioma	Drug: Valproic acid Drug: Bevacizumab	Texas Children’s Hospital + 4 others

NCT00890786	A study of bevacizumab therapy in patients with newly diagnosed high-grade gliomas and DIPG	Drug: Temozolomide Drug: Bevacizumab Drug: Irinotecan	Cincinnati Children’s Hospital Medical Center and Ann and Robert H. Lurie Children’s Hospital of Chicago

NCT01182350	Molecularly determinized treatment of DIPG	Drug: Bevacizumab Drug: Erlotinib Drug: Temozolomide	Dana Farber Cancer Institute + 22 others

NCT01189266	Vorinostat and radiation therapy followed by maintenance therapy with vorinostat in treating younger patients with newly diagnosed diffuse intrinsic pontine glioma	Drug: Vorinostat	Children’s Oncology Group (181 centers)

NCT01222754	Lenalidomide and radiation therapy in high grade gliomas or DIPG	Drug: Lenalidomide	National Institutes of Health Clinical Center

NCT01514201	Veliparib, radiation therapy, and temozolomide in treating younger patients with newly diagnosed diffuse pontine glioma	Drug: Veliparib Drug: Temozolomide	Texas Children’s Hospital + 10 others

NCT01922076	WEE1 inhibitor AZD1775 and local radiation therapy in treating children with newly diagnosed DIPG	Drug: WEE1 inhibitor AZD1775	Children’s Oncology Group Phase I Consortium (24 centers)

NCT02274987	Molecular profiling for individualized treatment plan for DIPG	Other: specialized tumor board recommendation	UCSF Benioff Children’s Hospital + 4 others

NCT02420613	Study of suberoylanilide hydroxamic acid (SAHA) with temsirolimus in children with DIPG	Drug: Vorinostat Drug: Temsirolimus	University of Texas MD Anderson Cancer Center

NCT02644460	Abemaciclib in children with DIPG or recurrent/refractory solid tumors	Drug: Abemaciclib	Children’s Hospital of Atlanta and Children’s Hospital of Colorado

NCT02992015	Gemcitabine in newly diagnosed diffuse intrinsic pontine glioma	Drug: Gemcitabine	Children’s Hospital of Colorado

NCT03396575	Brain stem gliomas treated with adoptive cellular therapy during focal radiotherapy recovery alone or with dose-intensified temozolomide (Phase I)	Biological: TTRNA-DC vaccines with GM-CSF + TTRNA-xALT with Td vaccine Drug: Cyclophosphamide + Fludarabine Lymphodepletive Conditioning Drug: Dose-Intensified TMZ	University of Florida

NCT03416530	ONC201 in pediatric H3K27M gliomas	Drug: ONC201	New York University and University of Texas MD Anderson Cancer Center

**Post-radiation therapy DIPG trials**			

NCT01130077	A pilot study of glioma associated antigen vaccines in conjunction with poly-ICLC in pediatric gliomas	Biological: HLA-A2 restricted glioma antigen peptides vaccine Biological: poly-ICLC	Children’s Hospital of Pittsburgh of UPMC

NCT01502917	Convection-enhanced delivery (CED) of 124I-8H9 for patients with non-progressive diffuse pontine gliomas previously treated with external beam radiation therapy	Radiation: Radioactive iodine-labeled monoclonal antibody 8H9	Memorial Sloan Kettering Cancer Center + Weill Cornell Medical College/New York Presbyterian Hospital

NCT01644773	Study of the combination of crizotinib and dasatinib in pediatric research participants with DIPG and high-grade glioma (HGG)	Drug: Crizotinib Drug: Dasatinib	St. Jude Children’s Research Hospital

NCT01837862	A phase I study of mebendazole for the treatment of pediatric gliomas	Drug: Mebendazole Drug: Temozolomide Drug: Bevacizumab Drug: Irinotecan	Cohen Children’s Medical Center of New York

NCT02343406	Evaluation of ABT-414 in children with high-grade gliomas (INTELLANCE 2)	Drug: ABT-414; Drug: Temozolomide	Children’s Hospital of Colorado, Dana Farber Cancer Institute, Stanford University Lucile Packard Children’s Hospital, UCSF Benioff Children’s Hospital

**Post-radiation therapy DIPG trials**			

NCT02607124	A phase I/II study of ribociclib, a CDK4/6 inhibitor following radiation therapy	Drug: Ribociclib	Cincinnati Children’s Hospital Medical Center

NCT02717455	Trial of panobinostat in children with diffuse intrinsic pontine glioma	Drug: Panobinostat	Stanford University and Lucile Packard Children’s Hospital + 9 others

NCT02742883	A study of atengenal and astugenal in diffuse intrinsic pontine glioma	Drug: Antineoplaston therapy (Atengenal + Astugenal)	Burzynski Clinic

NCT02960230	H3.3K27M peptide vaccine for children with newly diagnosed DIPG and other gliomas	Biological: K27M vaccine	UCSF Benioff Children’s Hospital + 10 others

NCT03086616	CED with irinotecan liposome injection using real-time imaging in children	Drug: Convection Enhanced Delivery of Nanoliposomal irinotecan (nal-IRI)	UCSF Benioff Children’s Hospital

NCT03330197	A study of Ad-RTS-hIL-12 + Veledimex in pediatric subjects with brain tumors or DIPG	Biological: Ad-RTS-hIL-12 Drug: Veledimex	Dana Farber Cancer Institute and Ann and Robert H Lurie Children’s Hospital of Chicago

NCT03355794	A study of ribociclib and everolimus following radiation therapy in children with newly diagnosed non-biopsied DIPG and RB + Biopsied DIPG and HGG	Drug: Ribociclib Drug: Everolimus	Cincinnati Children’s Hospital Medical Center

NCT03389802	Phase I study of APX005M in pediatric CNS tumors	Biological: APX005M	Memorial Sloan Kettering Cancer Center + 11 others

NCT03416530	ONC201 in pediatric H3K27M gliomas	Drug: ONC201	New York University and University of Texas MD Anderson Cancer Center

**Refractory or progressive DIPG trials**			

NCT01469247	DIPG reirradiation (reRT)	Radiation: Radiation therapy	University of Texas MD Anderson Cancer Center and Orlando Health

NCT01644773	Study of the combination of crizotinib and dasatinib in pediatric research participants with DIPG and HGG	Drug: Crizotinib Drug: Dasatinib	St. Jude Children’s Research Hospital

NCT01884740	Intraarterial infusion of erbitux and bevacizumab for relapsed/refractory intracranial glioma in patients under 22	Drug: SIACI of Erbitux and Bevacizumab	Weill Cornell Medical College/New York Presbyterian Hospital

NCT02323880	Selinexor in treating younger patients with recurrent or refractory solid tumors or HGG	Drug: Selinexor	Children’s Oncology Group Phase I Consortium (22 centers)

NCT02343406	Evaluation of ABT-414 in children with HGG (INTELLANCE 2)	Drug: ABT-414 Drug: Temozolomide	Children’s Hospital of Colorado, Dana-Farber Cancer Institute, Stanford University Lucile Packard Children’s Hospital, UCSF Benioff Children’s Hospital

NCT02359565	Pembrolizumab in treating younger patients with recurrent, progressive, or refractory HGG, DIPG, or hypermutated brain tumors	Biological: Pembrolizumab	Children’s National Medical Center + 8 others

NCT02420613	Study of suberoylanilide hydroxamic acid (SAHA) with temsirolimus in children with DIPG	Drug: Vorinostat Drug: Temsirolimus	University of Texas MD Anderson Cancer Center

NCT02502708	Study of the IDO pathway inhibitor, indoximod, and temozolomide for pediatric patients with progressive primary malignant brain tumors	Drug: Indoximod Drug: Temozolomide	Children’s Hospital of Atlanta and Augusta University

NCT02644291	Phase I study of mebendazole therapy for recurrent/progressive pediatric brain tumors	Drug: Mebendazole	Johns Hopkins University School of Medicine and Johns Hopkins All Children’s Hospital

NCT02644460	Abemaciclib in children with DIPG or recurrent/refractory solid tumors	Drug: Abemaciclib	Children’s Hospital of Atlanta and Children’s Hospital of Colorado

NCT02684058	Phase II pediatric study with dabrafenib in combination with trametinib in patients with HGG	Drug: Dabrafenib Drug: Trametinib	Children’s National Medical Center, Dana-Farber Cancer Institute + other institutions

NCT02717455	Trial of panobinostat in children with diffuse intrinsic pontine glioma	Drug: Panobinostat	Stanford University and Lucile Packard Children’s Hospital + 9 others

NCT02742883	A study of atengenal and astugenal in diffuse intrinsic pontine glioma	Drug: Antineoplaston therapy (Atengenal + Astugenal)	Burzynski Clinic

**Refractory or progressive DIPG trials**			

NCT02885324	Pilot study of cobazantinib for recurrent or progressive high-grade glioma in children	Drug: Cobazantinib	Riley Hospital for Children at Indiana University Health

NCT03126266	Re-irradiation of progressive or recurrent DIPG	Radiation: re-irradiation	Alberta Children’s Hospital

NCT03155620	Pediatric MATCH: targeted therapy directed by genetic testing in treating pediatric patients with relapsed or refractory advanced solid tumors, non-Hodgkin lymphomas, or histiocytic disorders	Drug: Larotrectinib Drug: Erdafitinib Drug: Tazemetostat Drug: PI3K/mTOR Inhibitor LY3023414 Drug: Selumetinib Drug: Ensartinib Drug: Vemurafenib Drug: Olaparib	Children’s Oncology Group (80 centers)

NCT03250520	Application of palliative treatment in children with brain stem glioma and recurrent high-grade tumors in the central nervous system with the nanomaterial NPt-Ca	Drug: platinum acetylacetonate (1% wt) supported by sol-gel technology functionalized titania	Hospital Infantil de Mexico Federico Gomez

NCT03257631	A study of pomalidomide (CC-4047) monotherapy for children and young adults with recurrent or progressive primary brain tumors	Drug: Pomalidomide	Ann and Robert H. Lurie Children’s Hospital of Chicago, Baylor College of Medicine, Dana-Farber Cancer Institute, National Cancer Institute, Stanford University Cancer Center, University of Florida

NCT03387020	Ribociclib and everolimus in treating children with recurrent or refractory malignant brain tumors	Drug: Everolimus Drug: Ribociclib	Cincinnati Children’s Hospital Medical Center + 11 others

NCT03416530	ONC201 in pediatric H3K27M gliomas	Drug: ONC201	New York University and University of Texas MD Anderson Cancer Center

NCT03434262	Molecularly driven doublet therapy for recurrent CNS malignant neoplasms	Drug: Gemcitabine Drug: Ribociclib Drug: Sonidegib Drug: Trametinib Biological: Filgrastim Biological: Pegfilgrastim	St. Jude Children’s Research Hospital

*Terms of search “DIPG,” “High grade glioma,” and limited to North American pediatric trials*.

The common DIPG mutation in Histone 3.3 or 3.1 alters the distribution of the repressive trimethylation at position 27, which leads to transcriptional de-repression. Preclinically, researchers utilized histone deactelyase inhibitors to overcome this epigenetic mutation with good effect. This has led to the initiation of a number of phase I clinical trials with agents such as valproic acid, entinostat, and panobinostat (LHB589) for the treatment of children with recurrent or progressive DIPG. While the preclinical data for histone modifications has been very exciting (46–48), the concentrations achieved in humans for these inhibitors before excessive toxicity have limited their clinical activity (47).

The rapid improvements in our understanding of immune regulation have led to a number of new approaches in the treatment of DIPG under the label of immunotherapy. These include vaccines, checkpoint inhibitors, and cellular therapies (NK, T cells, macrophages). Our understanding of immune regulation in the brain may be different from that outside the central nervous system. Thus, approaches that have demonstrated dramatic responses in certain leukemias, lymphoma, and melanoma (49–52) remain to be proven in DIPG and other CNS tumors (53).

Given the demonstrated safety of biopsy in newly diagnosed DIPG as detailed above (23–26), there has been a push for use of biopsy and the molecular information gained from these procedures to improve up front therapy in these devastating tumors. The first North American clinical trial (NCT01172350) tested the feasibility of biopsy followed by molecular stratification based on MGMT promoter methylation, EGFR overexpression, and subsequent treatment stratification (25). Given the success of this pilot study, multiple upfront biopsy protocols are now underway and at least one active clinical trial utilizes a precision medicine approach whereby a specialized treatment recommendation is made based upon RNA expression analysis, whole exome sequencing, and predictive modeling following biopsy in newly diagnosed DIPG (NCT02264987).

Additional trials presently seek to expand the role of convection enhanced delivery in DIPG. One trial involves the direct infusion of the traditional chemotherapy agent, irinotecan (NCT03086616). Another study involves direct delivery *via* CED of the 124I-8H9 radioactive antibody into the tumor (NCT01502917). Both of these trials employ these interventions after standard radiation therapy but prior to progression.

Still other current clinical trials seek to explore the feasibility and role of intra-arterial treatment in DIPG. One group is evaluating the safety of intra-arterial melphalan in progressive DIPG (NCT01688401). Another group is investigating the safety of intra-arterial erbitux, an EGFR inhibitor, and bevacizumab in relapsed/refractory intracranial glioma including DIPG (NCT01884740).

Thus, as biologic understanding of DIPG and technology advance, a new wave of clinical trials has emerged (Table 1).

## Conclusion

For decades, DIPG has stubbornly remained a disease with abysmal outcomes. However, safe biopsy has lead to improved biologic understanding of these challenging tumors as distinct from adult high-grade gliomas. With this understanding, our treatment paradigms have evolved. No longer are we treating DIPG with the same interventions as adult high-grade gliomas and wondering why these approaches are not effective. Instead, we seek to exploit the biologic characteristics of DIPG and employ strategies, which circumvent the unique challenges of the blood–brain barrier in this location. While exciting, we have not yet seen this new molecular understanding translate into more effective therapy. In the future, using these strategies in combination as well as a move toward precision medicine targeting individual mutational profiles of each tumor may finally alter the outcomes in DIPG.

## Author Contributions

JC and MK were involved in the composition and editing of the manuscript.

## Conflict of Interest Statement

The authors declare that the research was conducted in the absence of any commercial or financial relationships that could be construed as a potential conflict of interest.
